# Nanocage-incorporated engineered destabilized 3'UTR ARE of ERBB2 inhibits tumor growth and liver and lung metastasis in EGFR T790M osimertinib- and trastuzumab-resistant and ERBB2-expressing NSCLC via the reduction of ERBB2

**DOI:** 10.3389/fonc.2024.1344852

**Published:** 2024-04-18

**Authors:** Chidiebere U. Awah, Joo Sun Mun, Aloka Paragodaarachchi, Baris Boylu, Martin Nzegwu, Hiroshi Matsui, Olorunseun Ogunwobi

**Affiliations:** ^1^ Department of Biological Sciences, Hunter College of The City University of New York, New York, NY, United States; ^2^ Joan and Sanford I. Weill Department of Medicine, Weill Cornell Medicine, Cornell University, New York, NY, United States; ^3^ Department of Chemistry, Hunter College, City University of New York, New York, NY, United States; ^4^ Ph.D. Program in Biochemistry, The Graduate Center of the City University of New York, New York, NY, United States; ^5^ Department of Pathology, University of Tulane School of Medicine, New Orleans, LA, United States; ^6^ Ph.D. Program in Chemistry, The Graduate Center of City University of New York, New York, NY, United States; ^7^ Department of Biochemistry, Weill Cornell Medical College, New York, NY, United States; ^8^ Hunter College for Cancer Health Disparities Research, Hunter College of The City University of New York, New York, NY, United States

**Keywords:** NSCLC EGFRT790M, ERBB2/HER2, osimertinib and trastuzumab resistance, engineered mRNA destabilization, mRNA overwriting, iron oxide nanocages (IO), lung and liver metastasis inhibition, nonsense mediated decay

## Abstract

Non-small cell lung cancer (NSCLC) caused more deaths in 2017 than breast cancer, prostate, and brain cancers combined. This is primarily due to their aggressive metastatic nature, leading to more fatal rates of cancer patients. Despite this condition, there are no clinically approved drugs that can target metastasis. The NSCLC with EGFR T790M-overexpressing HER2 shows the resistance to osimertinib and trastuzumab starting 10–18 months after the therapy, and thus prospects are grim to these patients. To target the recalcitrant ERBB2 driver oncogene, we developed two engineered destabilizing 3'UTR ERBB2 constructs that degrade the endogenous ERBB2 transcript and proteins by overwriting the encoded endogenous ERBB2 mRNA with the destabilizing message. When iron oxide nanocages (IO nanocages) were used as vehicles to deliver them to tumors and whole tissues in mice bearing tumors, it was well tolerated and safe and caused no genome rearrangement whereas they were integrated into genome deserts (non-coding regions). We achieved significant reduction of the primary tumor volume with desARE3'UTRERBB2-30, achieving 50% complete tumor lysis and inhibiting 60%–80% of liver metastasis, hepatomegaly, and 90% of lung metastasis, through ERBB2 downregulation. These constructs were distributed robustly into tumors, livers, lungs, kidneys, and spleen and mildly in the brain and not in the heart. They caused no abnormality in both short- and long-term administrations as well as in healthy mice. In summary, we accomplished significant breakthrough for the therapeutics of intractable lung cancer patients whose cancers become resistant and metastasize.

## Graphical Abstract

**(A–C)**. The illustration shows the primary tumors and liver and lung metastasis of the non-small cell lung cancer. **(B–D)**. Illustration shows the primary non-small cell lung cancer treated with the desARE3'UTRERBB2-3 and 30 + IO-nanocage complexes, which inhibited the primary tumors and lung and liver metastasis.

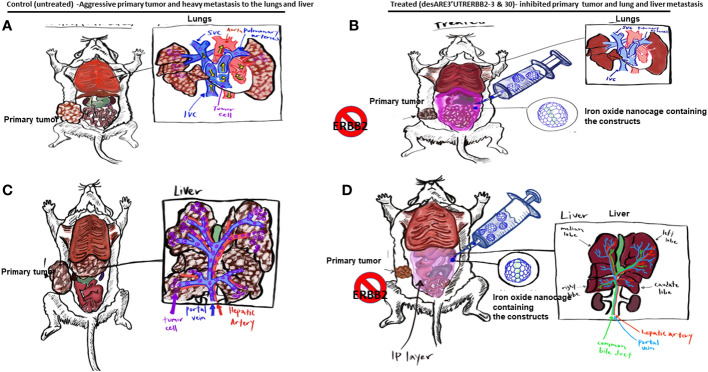

## Introduction

According to NCI SEER statistics, 53.1 per 100,000 men and women will develop lung and bronchial cancer per year, with a 5-year survival rate at 21.7% (1–3). Lung cancer caused more deaths in 2017 than breast, prostate, and brain cancers combined. The prevalence of lung cancer and its related mortality arises from the oncogenes that drive the malignant lung tissues to excessively proliferate and then metastasize to distant lymph nodes and onward to organs such as brain, liver, bones, and adrenal glands leading to death of the patient.

HER2/ERBB2 is a member of the subclass I receptor tyrosine kinase superfamily of ERBB/EGFR (epidermal growth factor receptor family) which consists of four members, namely, EGFR/ERBB1, ERBB2, ERBB3, and ERBB4 (4). Non-small cell lung cancer (NSCLC) with EGFR T790M is moderately responsive to first- and second-generation EGFR tyrosine kinase inhibitor (5–13). However, osimertinib which is a third-generation EGFR-TKI has been shown to be superior to erlotinib and gefitinib in a clinical trial AURA and FLAURA in lung cancer. Osimertinib is particularly effective for 60% of lung cancer patients who have EGFR mutation (T790M), but for the remaining 40%, there is no benefit and resistance start arising 10–18 months after the therapy. Resistance to osimertinib can arise in two ways, EGFR-dependent and EGFR-independent mechanisms. For the EGFR-dependent mechanism, it was found that mutation arises due to the following: MET amplification (15% patients), K-RAS and N-RAS (8% of patients), PI3KCA (7% patients), HER2 amp (5%–6% of patients), and MAPK1 and EGFR and BRAF. Moreso studies have shown that the treatment with osimertinib leads to increased HER2 surface expression and HER2 amplification. The EGFR-independent mechanism described the osimertinib resistance to be under the control of PTEN, TSC2, YES1, CTNNB1, FGF2, etc.

To address these problems, we have developed a new technology wherein we have engineered stable poly U rich elements on the 3'UTR of ERBB2 to unstable forms and destabilized the transcript and degraded the expression of ERBB2 protein. We used this technology to control ERBB2 in EGFR T790M HER2-overexpressing osimertinib- and trastuzumab-resistant NSCLC. This treatment significantly reduced the primary tumors and inhibited 80% of liver and lung metastasis in mice that received our constructs (14). This finding has provided a potential therapy to address this intractable end-stage drug-resistant NSCLC.

In this study, we hypothesized that the 3'UTR of oncogenes such as HER2 will be enriched with ARE-stabilizing elements, which stabilizes their oncogenic transcript and drives tumor aggressiveness, and therefore, if we change these stable elements to destabilizing elements by motif engineering and driven by mRNA de-capping promoter DCP1A, we can control the oncogene by degrading the oncogenic transcript and protein. We discovered that there are unique sequences on the poly U-rich elements (ARE) of the 3'UTR of mRNA that makes oncogenic HER2 transcript stable (15–17), and thus we engineered these elements to unstable forms and degraded HER2 across many HER2-driven cancer cell types both in wild-type and drug-resistant cancer models, which reduced primary tumor growth and inhibited metastasis (liver and lungs) *in vivo* in EGFRT790M HER2-positive osimertinib/trastuzumab-resistant NSCLC.

We developed two constructs desARE3'UTRERBB2-3 and 30, effective for destabilizing the ERBB2 transcript and degrading the protein, and ERBB2-dependent kinases WNK1, and YES1 in EGFR T790M NSCLC. The loss of ERBB2 led to the loss of cell viability. The degradation of ERBB2 is through upregulated nonsense-mediated decay proteins UPF3B, CNOT1, and XRN1 (18–21). In two independent *in vivo* studies, we achieved inhibition of primary tumor, inhibition of liver and lung metastasis, and control of hepatomegaly. The desARE3'UTRERBB2-3 and 30 are specific to ERBB2 and do not affect normal cells. We found no weight changes, blood dyscrasias, and electrolyte, renal, or liver malfunctions. The desARE3'UTRERBB2-3 and 30 were systemically biodistributed.

## Materials and methods

### Cell culture

The NCI H1975, HCC827, and NCIH460 cells were cultured in RPMI supplemented with the 10% FBS and 5% antibiotics. Cells were grown until they are 80% confluent before use.

### Engineering the destabilized 3'UTR ERBB2 constructs

The details of the engineering of the ERBB2 3'UTR constructs have been exhaustively described in the Awah et al., 2023 Frontiers Genetics. Briefly, we identified the poly U mRNA stabilizing sequences on the 3'UTR of ERBB2 across different ERBB2-driven cancers. We then generated a modular designed synthetic construct driven by the DCP1A promoter in which all the stable poly U sequences were changed to unstable forms. The synthetic construct was cloned either by sticky end ligation using BamH1 and BstB1 sites or by Gibson assembly to the receiving vector (pLenti-CMVSP6-nEGFP-SV40-PURO, Addgene #138364). They were used to transform competent E.coli, and the clones picked were miniprepped and confirmed by Sanger Sequencing. For generation of high-yield plasmid DNA containing the constructs for animal experiment, we used the Maxi Prep kit from Zymo Research (cat no.: D4203).

### Animal study

To perform the animal studies, we obtained the institutional IACUC approval. There were 25 NSG female mice ordered from the Jackson Laboratory. The mice were received and allowed to acclimatize according to institutional protocol. NCI-H1975 cells were cultured in RPM1; when at 80% confluency, the cells were harvested and washed in PBS and counted. Five million cells in 0.1 mL were implanted on the flank of the animals. After 35 days, huge tumors were engrafted, and on the 36th day, the animals were randomized into five mice per cage into five cages with equal distribution of tumor size (600 mm^3^–800 mm^3^) (**Supplementary Figure 1C**). On day 37, we started intraperitoneal administration of the iron oxide plus the engineered destabilized construct at 20 µg 12-hourly every 2 days per week between days 37 and 49. On day 50, we observed a dosing break and resumed from days 51 to 77 12-hourly every 2 days per week. At 77 days, the experiment ended. We performed complete necropsy and sent the organs to the core pathology group of the Memorial Sloan Kettering Cancer Institute for histological analysis and hematoxylin and eosin (H&E) staining. The expert pathologist was blinded to the experimental details.

### IO-nanocage packaging of the engineered destabilized construct

3,4-Dihydroxyhydrocinnamic acid (DHCA) by Alfa Aesar, manganese(II) acetate, oleylamine, oleic acid, and iron(II) perchlorate were purchased from Sigma-Aldrich. p-Xylene, 1-ethyl-3-[3-(dimethylamino)propyl]carbodiimide (EDC), and N-hydroxysuccinimide (NHS) were purchased from Thermo Scientific. Tetrahydrofuran (THF), dimethyl sulfoxide (DMSO), ferric chloride hexahydrate, and phosphate-buffered saline (PBS) were purchased from Fisher Scientific. Cy7.5 dye was purchased from Lumiprobe.

### Generation and structure analysis of DNA plasmid-IO-nanocage complexes

The IO nanocages were synthesized by a galvanic reaction as manganese ions of template Mn_3_O_4_ nanocubes are replaced by iron ions to form a cage structure with a hollow center, as shown in previous publications (22–24). Water-soluble IO nanocages were engineered by capping with 3-(3,4-dihydroxyphenyl) propionic acid (DHCA) before loading the DNA plasmids containing the destabilized ERBB2 constructs as well as vector controls only. DNA plasmids were mixed with IO nanocages with the mass ratio of 1:1 for the complexation based on the concentration of DNA plasmids needed for the sufficient efficacy with respect to the optimized dosage of IO-nanocage concentration for *in vivo* experiments.

### Hematoxylin and eosin staining and immunohistochemistry

The lung and liver H&E was done by an expert pathologist of the Memorial Sloan Kettering Cancer Center, Core Pathology Laboratory New York. BB performed ERBB2 immunohistochemistry (IHC) and primary tumor H&E according to standard protocol. After sacrifice, tumor, spleen, liver, kidney, lung, heart, and brain samples obtained from mice *via* dissection were fixed in formalin and embedded in paraffin. Sections of 4-μm thickness were stained with hematoxylin and eosin (H&E) and IHC antibodies.

HE and IHC staining was performed as previously described (25). Briefly, samples of the tumors, livers, brains, spleens, kidneys, and lungs were fixed, stained with HE, and visualized under an optical microscope. HE staining enabled determination of the morphological features of the tumors. For IHC staining, slides were deparaffinized, dehydrated, and blocked. The slides were blocked with mouse serum for 30 min and incubated with HER 2 antibody (1:500) (Proteintech, 18299-1-AP), were performed at 4°C overnight. All the buffer and reagents used in IHC were used from IHC Prep & Detect Kit for Mouse Primary Antibody (Proteintech, PK10018) according to manufacturer’s instructions.

Next, the slides were incubated with the secondary antibody, stained with diaminobenzidine substrate, and counterstained with hematoxylin. Lastly, all samples were analyzed *via* optical microscope.

### Prussian blue staining and detection of IO-nanocage in tissues

The Prussian Blue Staining on the tissue slides with and without the treatment of ERBB2-incorporated IO nanocages was performed as follows. First, the tissue slides were deparaffinized with xylene for 10 min, 100% ethanol, for 9 min, 95% ethanol for 6 min, and 70% ethanol for 3 min, and lastly hydrated with Milli-Q water for 5 min. Then, the tissue slides were placed in the Working Iron Staining Solution (Sigma-Aldrich) for 30 min as the Working Iron Stain Solution was prepared by mixing equal volumes (100 mL) of potassium ferrocyanide solution and hydrochloric acid solution. The tissue slides were then collected and rinsed thoroughly in Milli-Q water. The tissue slides were then placed in the working pararosaniline solution (Sigma-Aldrich) for 3 min–5 min as the working pararosaniline solution was prepared by mixing 1 mL of pararosaniline solution with 50 mL of Milli-Q water. After the tissue slides were collected and rinsed thoroughly in Milli-Q water, the tissue slides were then rapidly dehydrated through a series of alcohol treatment and cleaning with xylene as stated above before mounting. One drop of Permount Mounting Media (Fisher Scientific) was placed on the tissue slide, and a cover slip was placed on top of the tissue slide for imaging.

### Pathological scoring

Reporting of cancer cell lysis, mitotic counts, histological subtyping, and ERBB2 grading were done according to the following protocols (26, 27). Cell lysis was reported using high-resolution microscopy as well as observing the appearance of disrupted cell membranes and naked nuclei under high power resolution. Nottingham’s prognostic index was used to grade the number of mitoses in breast cancer 1, whereas histological typing was done according to the degree of differentiation, with poorly differentiated cells forming no glands and growing in sheets.

ERBB2 grading was considered positive if at least 30% of tumor cells exhibited 3+ cell membrane staining, and a borderline result was given when at least 10% of cells showed 2+ cytoplasmic membrane staining.

### Quantitative PCR

To validate the sensitivity and specificity of detecting the engineered destabilized 3'UTR of ERBB2 from the genomic DNA of the tumors from treated animals. We extracted the gDNA of the tumors according to standard protocol using the Qiagen kit. We measured the concentration of the DNA by nanodrop. To determine the sensitivity, specificity, and limit of detecting the constructs, we performed quantitative PCR from the extracted gDNA from the tumor using a serial dilution of 1, 1:10, and 1:1,000 assaying for the expression of the construct as well as the housekeeping gene GAPDH with primers that recognize only the engineered destabilized 3'UTR of ERBB2 and primers targeting the exons of GAPDH.

### Targeted sequencing

To detect the integration site of the engineered destabilized constructs, we performed a targeted sequencing of the gDNA obtained from the tumors of the treated animals. We used a set of primers that recognizes the engineered destabilized 3'UTR ERBB2 constructs and the RFP marker on the vector in the genomic DNA, thus validating that they reliably detected the construct and the vector.

### Biodistribution analysis

To detect the biodistribution of the constructs across the tissues and organs of the treated mice, we extracted genomic DNA from the tumors, spleens, livers, lungs, hearts, and brains of the treated animals. We performed PCR with primers that only detect the engineered destabilized 3'UTR ERBB2 constructs. The amplified products were resolved on 2% agarose gels and imaged.

### Statistical analysis

All the experiments done were performed in replicates, starting with the animal experiment. A total of 25 NSG female mice were used at five mice per group per cage. The groups were IO-nanocage only, IO-nanocage+vector, desARE3'UTRERBB2-1+IO-nanocage, desARE3'UTRERBB2-3+Nanocage, and desARE3'UTRERBB2-30+IO-nanocage. A two-tailed t-test was used to determine statistical significance between the controls and the different treatment groups.

### GraphPad prism

All graphs plotted were drawn with the GraphPad Prism software, and all statistical significance analysis was done with the same tool.

## Results

### IO nanocage delivered engineered destabilized ERBB2 constructs inhibits primary EGFR T790M HER2+ osimertinib-resistant NSCLC

**Figure 1:**
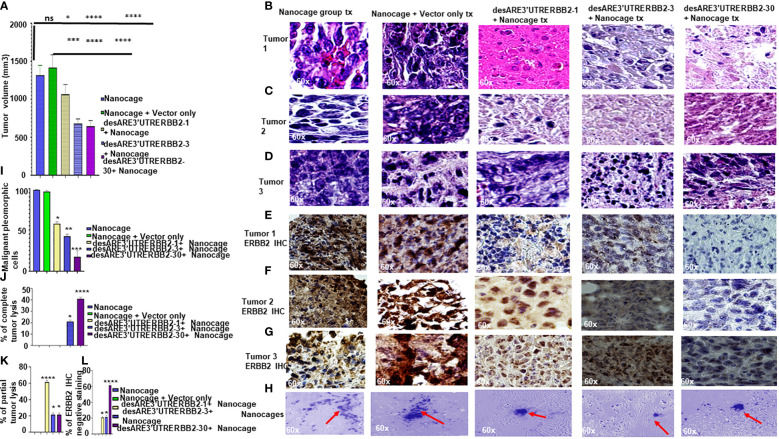
The engineered destabilized 3'UTR ERBB2 constructs inhibits tumor growth with complete tumor lysis *via* downregulation of ERBB2 *via* delivery by IO-nanocage vehicles. **(A)** Bar charts show the tumor volume of the control groups (IO-nanocage and IO-nanocage+Vector) and the treated groups the desARE3'UTR ERBB2-1, 3, and 30-treated groups. Two tailed T-test (IO nanocage only versus IO nanocage + vector p = ns; *p = 0.01 nanocage only vs. desARE3'UTRERBB2-1; ****p = 0.0003 IO nanocage only vsdesARE3'UTRERBB2-3; ****p = 0.0003 nanocage only vs. desARE3'UTRERBB2-30), (***p = 0.001 IO nanocage + vector vs. desARE3'UTRERBB2-1; ****p = 0.00033 IO nanocage + vector vs. desARE3'UTRERBB2-3; ****p = 0.00032 IO nanocage + vector vs. desARE3'UTRERBB2-30). **(B–D)** Panel shows images of H&E staining of the three tumors from the control and treatment groups. **(E–G)** Panel shows images of ERBB2 IHC staining (brown) and DAPI (nuclei-blue staining) of the three tumors each from the control and treatment groups. **(H)** Panel shows images of IO nanocages (Prussian blue) in tumor tissues of the control and treated groups. **(I)** Bar chart shows percentage of malignant pleomorphic cells per field of the control and treated groups. Two tailed T-test (p = ns IO nanocage only versus IO nanocage + vector, *p = 0.01 IO nanocage only versus desARE3'UTRERBB2-1, *p = 0.003 IO nanocage only vs. desARE3'UTRERBB2-3, **p = 0.00022 IO nanocage only versus desARE3'UTRERBB2-30). **(J)** Bar chart shows the percentage of complete tumor lysis in the control and treated groups. Two tailed T-test (*p = 0.01 IO nanocage only vs. desARE3'UTRERBB2-3, ****p = 0.000014 nanocage only versus desARE3'UTRERBB2-30). **(K)** Bar chart shows the percentage of partial tumor lysis in the control and the treated groups. Two tailed T-test (****p = 0.00003 nanocage only versus desARE3'UTRERBB2-1, *p = 0.01 IO nanocage only vs. desARE3'UTRERBB2-3 and 30). **(L)** Bar chart shows the percentage of the ERBB2 IHC-negative staining in the control and treatment groups. Two tailed T-test (*p = 0.02 IO nanocage only vs. desARE3'UTRERBB2-1, *p = 0.013 IO nanocage only versus desARE3'UTRERBB2-3, ****p = 0.00045 nanocage only vs. desARE3'UTRERBB2-30). ns, not significant.

To determine if the engineered destabilized 3'UTR ERBB2 constructs packaged into the IO nanocage (22–24, 28, 29) inhibit the primary tumor, we implanted 25 NSG mice with five million NCI-H1975 cells into the flank; after 35 days of tumor engraftment, we randomized the mice into five mice per cage on the day 36 (**Supplementary Figure 1A**). Starting from days 37 to 43, we administered desARE3'UTRERBB2-1, 3, and 30 as IO nanocage complexed intraperitoneally at 20 µg/0.1 mL every 12 h for every 2 days per week till day 77 (**Supplementary Figure 1A**). There were dosing breaks at days 49–50. We weighed the animals daily and measured the tumor size with calipers and noted body conditions. We found no change in body weight in the treated animals, meaning no toxicity (**Supplementary Figure 1B**). The constructs desARE3'UTRERBB2-3,30 significantly inhibited tumors (**Figures 1A–H**, ****P=0.0001). The construct desARE3’UTRERBB2-30 achieved a very significant reduction of malignant pleomorphic cells, an 80% reduction as compared with the controls (**Figures 1B–D, I**). The desARE3'UTRERBB2-30 achieved 40% complete tumor lysis, a 20% partial tumor lysis, and 60% ERBB2 negative staining by IHC (**Figures 1E–G, J–L**). The desARE3'UTRERBB2-1 and 3 achieved variable complete (20% desARE3'UTRERBB2-3) and partial (60% and 20%) tumor lysis respectively, with 20% ERBB2-negative IHC staining (**Figures 1E–G, J–L**). Both empty IO nanocages and construct-nanocage complexes were detected across all tumors by Prussian blue stain (**Figure 1G**). Taken together, these data validate the proof-of-concept study that we presented in the previous works (14).

### The destabilized construct inhibited 80% liver metastasis in EGFR T790M HER2+ osimertinib-resistant NSCLC

**Figure 2:**
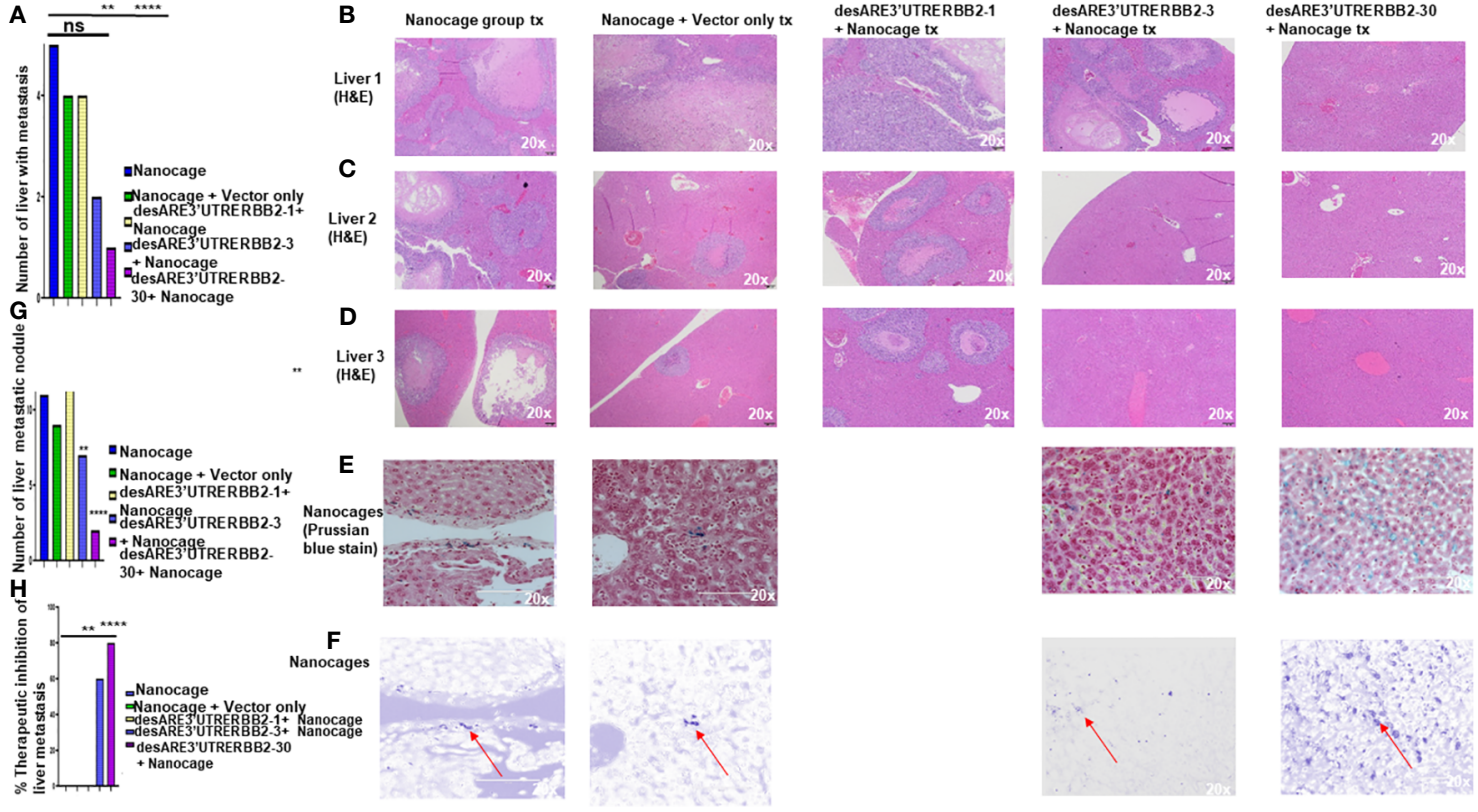
The destabilized 3'UTR ERBB2 constructs inhibits liver metastasis *via* IO-nanocage delivery. **(A)** Bar chart shows the number the livers with metastasis in the controls (IO nanocage only, IO nanocage + vector) and the treated groups desARE3'UTRERBB2-1+nanocage, desARE3'UTRERBB2-3+IO-nanocage, desARE3'UTRERBB2-30 + IO nanocage. Two tailed T-test (p = ns IO nanocage only vs. IO-nanocage + vector, p = ns IO nanocage only vs. desARE3'UTRERBB2-1, **p = 0.002 IO nanocage only versus desARE3'UTRERBB2-3, ***p = 0.00013 IO nanocage only vs. desARE3'UTRERBB2-30). **(B–D)** Panel shows images of H&E staining of three livers from the control and treated groups. **(E, F)** Panel shows images of livers from the control and treated groups stained with Prussian blue stain for IO nanocage detection and identification. **(G)** Bar chart shows the number of metastatic liver nodules in the control and treated groups. Two tailed T-test (p = ns nanocage only vs. nanocage + vector, p = ns nanocage only vs. desARE3'UTRERBB2-1, **p = 0.003 nanocage only vs. desARE3'UTRERBB2-3, **p = 0.00025 nanocage only vs. desARE3'UTRERBB2-30). **(H)** Bar chart shows percentages of therapeutic inhibition of liver metastasis in the control and treated groups. Two tailed T-test (p = ns IO nanocage only versus IO nanocage + vector, p = ns IO-nanocage only vs. desARE3'UTRERBB2-1, p = 0.0013 IO nanocage only versus desARE3'UTRERBB2-3, ***p = 0.00018 IO-nanocage only versus desARE3'UTRERBB2-30). ns, not significant.

Metastasis is the number one killer of cancer patients, and there are no therapies targeting it. To determine that our constructs inhibited liver metastasis, we performed necropsy and assessed first the gross pathology of the liver to ascertain visible liver metastasis (**Supplementary Figure 2**) and then we sectioned the livers and stained the tissues with H&E. We found that the desARE3'UTRERBB2-30-treated group had significantly the lowest number of livers with metastasis and lowest number of livers with metastatic nodules and achieved 80% therapeutic inhibition of liver metastasis (**Figures 2A–H**). The desARE3'UTRERBB2-3 achieved 60% therapeutic inhibition of liver metastasis (**Figure 2H**). Taken together, this demonstrates that the therapeutic efficacy of the constructs in inhibiting the primary tumor and metastatic liver nodules.

### Engineered destabilized 3'UTR of HER2 inhibits lung metastasis

**Figure 3:**
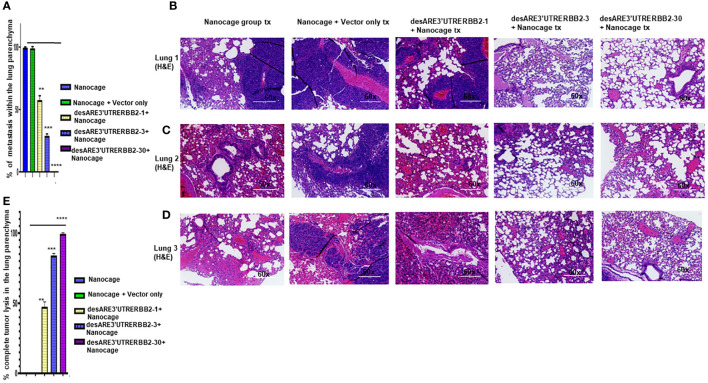
The destabilized 3'UTR ERBB2 inhibits lung metastasis *via* IO-nanocage delivery. **(A)** Bar charts show percentages of metastatic lesions within the lung parenchyma. Two-tailed T-test (p = ns IO nanocage only versus IO nanocage + vector, **p = 0.0025 IO nanocage only versus desARE3'UTRERBB2-1, ***p = 0.00042 IO nanocage only versus desARE3'UTRERBB2-30, and **p = 0.000013 IO nanocage only versus desARE3'UTRERBB2-30). **(B–D)** Panel shows images of H&E staining of three lungs from each experimental group, the controls, and treated groups. **(E)** Bar charts show percentages of complete tumor lysis within the lung parenchyma. Two tailed T-test (p = ns IO nanocage only versus IO nanocage + vector, **p = 0.002 IO nanocage only versus desARE3'UTRERBB2-1, **p = 0.00012 IO nanocage only versus desARE3'UTRERBB2-3, ****p = 0.000035 IO nanocage only versus desARE3'UTRERBB2-30).

The NCI-H1975 EGFR T790M non-small cell lung cancer is highly metastatic. We explored if the treatment with the novel engineered destabilized 3'UTR of ERBB2 inhibited the lung cancer metastasis. We found that the desARE3'UTRERBB2-30 constructs completely inhibited the lung metastasis (**Figures 3A–D**, **Supplementary Figures 3**, **4**). The inhibition of the lung metastasis by desARE3'UTRERBB2-30 is followed by desARE3'UTRERBB2-3. desARE3'UTRERBB2-30 achieved complete tumor lysis of the metastatic tumors in the lung parenchyma, and this is followed by parenchyma (**Figure 3E**). Taken together, we show that desARE3'UTRERBB2-30 is very effective in inhibiting and destroying metastatic tumors in the lung parenchyma of the treated animals.

### IO-nanocages delivered the constructs to the tumors, liver, lungs, spleen, and kidney of the treated animals

**Figure 4:**
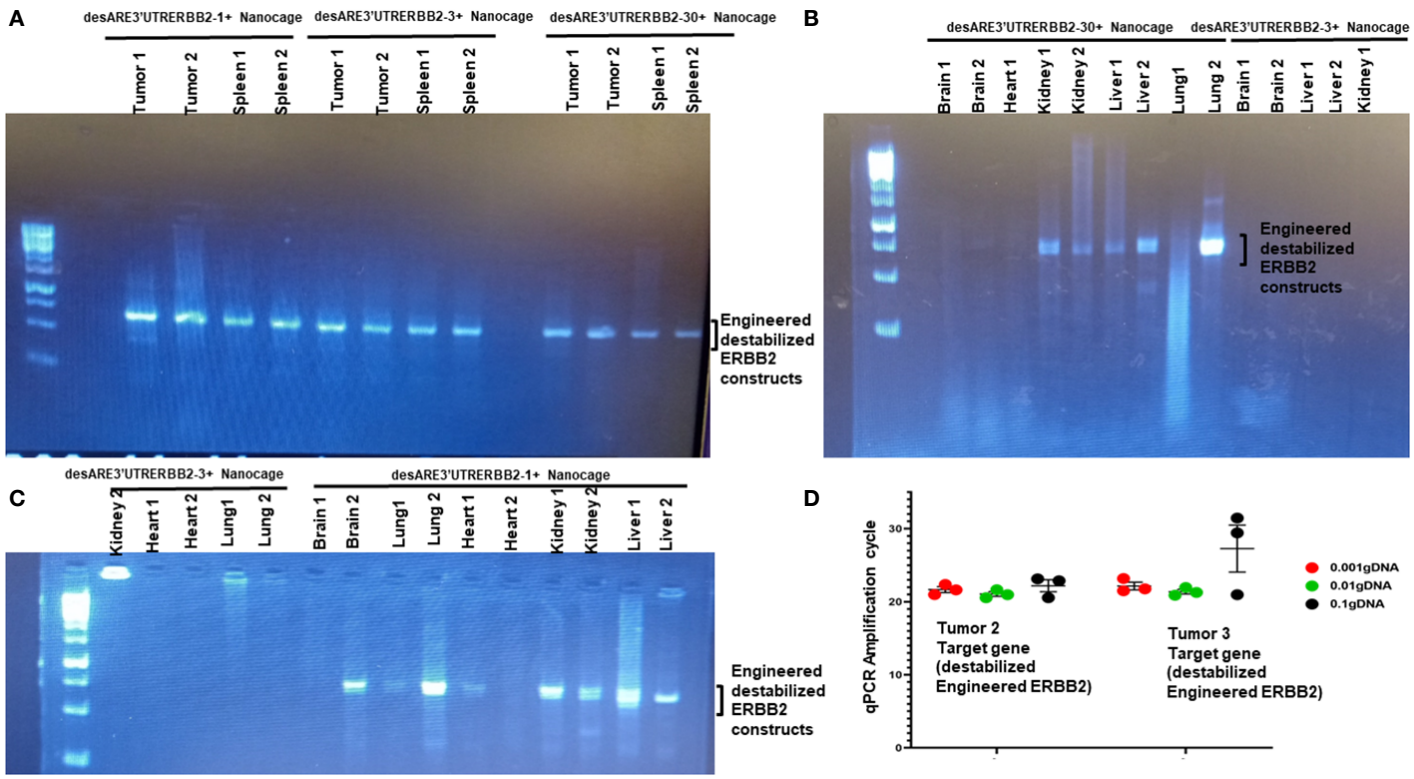
Biodistribution of IO-nanocage–construct complexes in the tumors, spleens, lungs, kidneys, and livers. **(A)** PCR gel image shows the engineered destabilized 3'UTRERBB2-1, 3, and 30 in the tumors and spleens from the treated animals. **(B)** PCR gel image shows the engineered destabilized 3'UTRERBB2-30 in the two kidneys, two lungs, and two livers. **(C)** PCR gel image shows the engineered destabilized 3'UTRERBB2-1 in the brain, lung, kidneys, and livers. **(D)** Chart shows the qPCR amplification cycle of the engineered destabilized 3'UTRERBB2 constructs from tumor genomic DNA in serial dilutions of 1, 1:10, and 1: 1,000.

To understand the biodistribution of the engineered destabilized ERBB2 constructs in the treated animals, we found that iron oxide nanocages were in the stellate hepatic cell as well as in the intrahepatic cell space of Disse and even following the patterns of liver parenchyma architecture (**Figure 2E**, desARE3’UTRERBB2-30+Nanocage). We used primers that are designed to specifically amplify the synthetic engineered destabilized ERBB2. We identified the engineered destabilized 3'UTR ERBB2 constructs in the tumors, spleens, kidneys, livers, and lungs of the treated animals (**Figures 4A–C**). We did not find the constructs in the brain and heart of the treated animals (**Figures 4B, C**). Only the destabilized 3'UTRERBB2-1 is expressed minimally expressed in the heart (**Figure 4C**). Next, we investigated the transcript level of the engineered destabilized ERBB2 constructs. We performed a quantitative polymerase chase reaction for the constructs on the tumor samples of the treated animals, and we found that the desARE3'UTRERBB2 transcript level is high (**Figure 4D**).

### The destabilized ERBB2 constructs integrated safely into non-coding regions

To ascertain the safety and genome integration site of the constructs in the treated animals, we performed targeted sequencing of the tumors using primers that identify only the engineered destabilized ERBB2 constructs and the RFP sequences in the vector (**Supplementary Figure 5A**). We found the constructs were integrated in the non-coding regions of chromosomes 1, 7, and 10 (**Supplementary Figure 5B**). This finding supports the safety profile as well as the ability of constructs not to cause genome rearrangements, as described previously (14). To validate that the housekeeping gene expression level is not perturbed by the constructs, we performed a head-to-head qPCR for ActB and RFP in the vector in the tumor samples and found that the ActB expression is higher than the RFP, indicating that the housekeeping genes for normal cellular functions are optimal (**Supplementary Figure 5C**).

### IO-nanocage-construct complexes delivered to the healthy animal with no tumors caused no vital organ damage

To prove the safety of the constructs to healthy non-tumor-bearing animals, we administered the constructs to animals intraperitoneally for 3 months. We found no gross organ abnormality across all the organs of the animals including the muscle tissues (**Supplementary Figures 6A, B**). The animals had no weight loss. Thus, here we showed that the constructs are safe and well tolerated.

## Discussion

Lung cancers have remained the leading cause of cancer death worldwide. Resistance to the tyrosine kinase inhibitor is rampant. We have demonstrated here that the recalcitrant ERBB2 signal driving the EGFR T790M non-small cell lung cancer can be degraded by the engineered destabilized 3'UTR of ERBB2. We show that the desARE3'UTRERBB2-30 constructs packaged into IO nanocages achieved 50% therapeutic inhibition of the tumor, 80% malignant cell reduction, 50% complete tumor lysis, and 60% complete reduction of ERBB2 IHC (**Figures 1A–L**).

Furthermore, the construct desARE3'UTRERBB2-30 achieved 80% therapeutic inhibition of liver metastasis (**Figure 2**) and 90% inhibition of lung metastasis with complete tumor lysis in the lung’s parenchyma (**Figure 3**). The IO nanocages delivered the constructs into the tumors, spleen, lung, livers, and kidneys (**Figure 4**), and the released constructs were integrated into the non-coding regions of the genome (**Supplementary Figure 5**). We found no evidence of weight loss in both the treated tumor and non-tumor bearing animals, which strongly suggests that it is well tolerated and safe. The findings presented here support the proof of concept published previously (14). To study the applicability of the desARE3'UTRERBB2-30 constructs in NSCLC with diverse genetic background, we used HCC827 (EGFR del 19 osimertinib resistant, ERBB2 expressing NSCLC) and NCIH460 (EGFR- and ERBB2-negative KRAS-positive NSCLC) in dose-dependent comparison with standard-of-care trastuzumab deruxtecan (**Supplementary Figure 7**). desARE3'UTERBB2-30 outperformed the standard-of-care trastuzumab deruxtecan in HCC827 NSCLC (**Supplementary Figure 7**).

Previously, the biodistribution and clearance of the exact same size of non-hollow superparamagnetic iron oxide nanoparticles were studied thoroughly. For the biodistribution, 20-nm IO nanoparticles in the same of the IO nanocage in this report were accumulated mostly in the liver (2.5 mg/g of organ at a dose of 50 mg/kg body weight) whereas heart, brain, and lung showed no significant accumulation after 24 h of injection. This value was equivalent to approx. 6.5% liver weight/per total weight ratio, whereas in the case of the spleen, the ratio was around 0.41% spleen weight per total weight ratio (30). The mechanism of biodegradation of magnetic nanoparticles is believed to be analogous to the metabolism of ferritin, which is digested by lysosomal enzymes to release iron ions (31). For the clearance mechanism, iron oxide magnetic nanoparticles can be removed by renal clearance (32) or by MPS (phagocytic cells in the blood, tissues, lymph nodes, etc.) (33). In fact, intravenously administered magnetic nanoparticles are captured by macrophages, mainly of the liver and spleen, and then digested in the acidic environment of the lysosomes (34), and the degradation of MNPs occurs faster in the liver macrophages than in the spleen, as the former contains more iron-storing proteins (35). Thus, the clearance and degradation mechanism of IO nanoparticles have been well studied, and the biosafety of IO nanoparticles in the size range of IO nanocages is quite high in general based on these literatures. Therefore, we expect that IO nanocages will behave similarly in the body and will be highly safe (or IO nanocages could be even safer due to the hollowness (i.e., less Fe content, less toxic).

Due to censorship of the tumor by the animal facility, this work lacked the survival study as well as relatively small sampling number of *in vivo* studies. Although this experiment is to validate the earlier published data, in future we will revalidate the data again in a larger cohort of mice that are immune competent (NSG-SGM3) donor balanced with equal distribution of sex including both male and female mice.

Taken together, we have presented here that the engineered destabilized 3'UTR ERBB2-30 delivered by IO nanocages inhibited the drug-resistant EGFR T790M NSCLC with 50% complete tumor lysis, 60% inhibition of ERBB2 protein, 50% therapeutic inhibition of tumor, and 80% reduction of malignant cells. These IO-nanocage–construct complexes were well tolerated, were systemically distributed, and caused no organ damage both to the tumor bearing and healthy mice. This novel drug and the delivery package represent a new paradigm to target drug-resistant cancers that are recalcitrant to available therapy.

## Data availability statement

The original contributions presented in the study are included in the article/**Supplementary Material**. Further inquiries can be directed to the corresponding authors.

## Ethics statement

The animal study was approved by OO/Weill Cornell Medicine. The study was conducted in accordance with the local legislation and institutional requirements.

## Author contributions

CA: Conceptualization, Data curation, Formal analysis, Investigation, Methodology, Validation, Visualization, Writing – original draft, Writing – review & editing. JS: Investigation, Writing – review & editing. AP: Investigation, Writing – review & editing. BB: Investigation, Writing – review & editing. MN: Formal analysis, Writing – review & editing. HM: Funding acquisition, Investigation, Resources, Supervision, Visualization, Writing – review & editing. OO: Conceptualization, Funding acquisition, Project administration, Resources, Supervision, Writing – review & editing.

## Supplementary material

Supplementary Figure 1 Scheme of the animal experiment. **(A)** Diagrammatic representation of the animal experiment starting with tumor implantation, engraftment, tumor randomization and dosing schedule. **(B)** Chart shows the plot of the daily weight of the animals bearing tumors, the controls, and the treatment groups. **(C)** Chart shows the daily tumor volume of the controls and treatment groups starting from day 0 of randomization to day 32 of the end of treatment. Two tailed t-test (**** p=0.000817962 Nanocage +Vector vs. desARE3’UTRERBB2-1, *****p=5.94e^-05^ IO-nanocage + Vector versus desARE3’UTRERBB2-3,*****p=4.17182e^-05^ IO-nanocage + Vector versus desARE3’UTRERBB2-30).

Supplementary Figure 2 Gross pathology of the livers of the control and treated groups **(A)** Images show the gross pathology of livers in the controls and treated groups with visible hepatomegaly and large metastatic liver nodules in the controls.

Supplementary Figure 3 H& E staining of the lung tissues from control and treated groups. **(A-C)** Panel of images shows the H & E staining of the lung tissues from the IO-nanocage only, IO-nanocage+ vector and desARE3’UTRERBB2-1 + IO-nanocages.

Supplementary Figure 4 H& E staining of the lung tissues from the desARE3’UTRERBB2-3 and 30 treated groups. **(A, B)** Panel of images shows the H&E staining of the lung tissues from the treated groups of desARE3’UTRERBB2-3 + IO-nanocages and desARE3’UTRERBB2-30 + IO-nanocages.

Supplementary Figure 5 Identification of genomic locations of the destabilized 3’UTRERBB2. **(A)** The schematic representation of the vector with the constructs and arrows (purple) represents the ERBB2 reverse primers and blue the RFP forward primers. **(B)** The circos plot shows the genomic localization of the engineered destabilized 3’UTR of ERBB2 on the intergenic regions of the chromosomes 1, 7 and 10. C. Bar charts show the qPCR amplification cycle of the house keeping gene ActB in comparison with the RFP in the vector in two tumors treated with the desARE3’UTRERBB2-30.

Supplementary Figure 6 Gross pathology images of the normal healthy female NSG mice (i.e., non-tumor bearing mice) treated with the desARE3’UTRERBB2-3 and 30 for 3 months. **(A, B)** Panel of images shows the gross images of the muscles, brains, livers, lungs, hearts, kidneys and spleens treated with the engineered destabilized 3’UTRERBB2-3 and 30.

Supplementary Figure 7 Dose response curve showing the viability dose dependent titration of desARE3’UTRERBB2-30 in Osimertinib resistant NSCLC HCC827 and NCIH460 in comparison with the standard of care trastuzumab deruxtecan. **(A)** Graph shows drug dose response of curve desARE3’UTRERBB2-30 and trastuzumab deruxtecan in NSCLC HCC827 and NCIH460.
