# Supplementary figures and images for: Nanocage-incorporated engineered destabilized 3'UTR ARE of ERBB2 inhibits tumor growth and liver and lung metastasis in EGFR T790M osimertinib- and trastuzumab-resistant and ERBB2-expressing NSCLC via the reduction of ERBB2
Source: Front Oncol. 2024 Apr 18;14:1344852. doi: 10.3389/fonc.2024.1344852 (PMC11063227; doi:10.3389/fonc.2024.1344852)

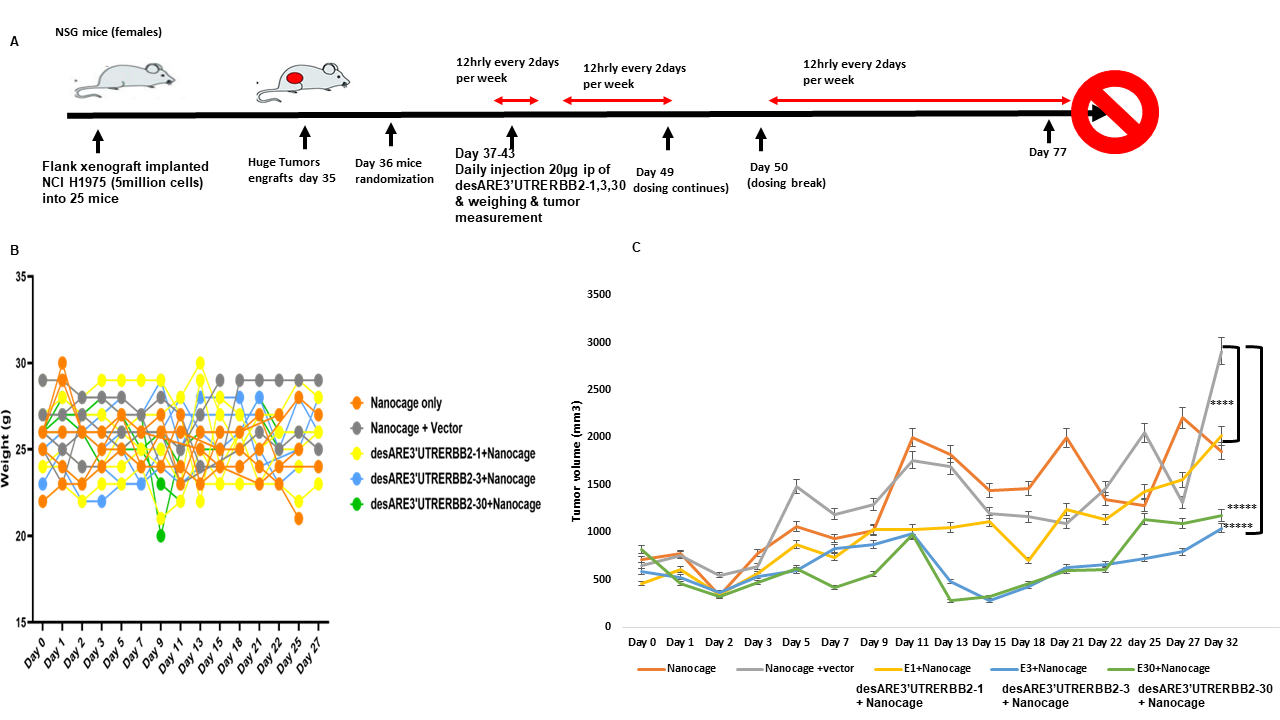

Supplement: Supplementary Figure 1 — Scheme of the animal experiment. (A) Diagrammatic representation of the animal experiment starting with tumor implantation, engraftment, tumor randomization and dosing schedule. (B) Chart shows the plot of the daily weight of the animals bearing tumors, the controls, and the treatment groups. (C) Chart shows the daily tumor volume of the controls and treatment groups starting from day 0 of randomization to day 32 of the end of treatment. Two tailed t-test (**** p=0.000817962 Nanocage +Vector vs. desARE3’UTRERBB2-1, *****p=5.94e-05 IO-nanocage + Vector versus desARE3’UTRERBB2-3,*****p=4.17182e-05 IO-nanocage + Vector versus desARE3’UTRERBB2-30). [file Image_1.tif]

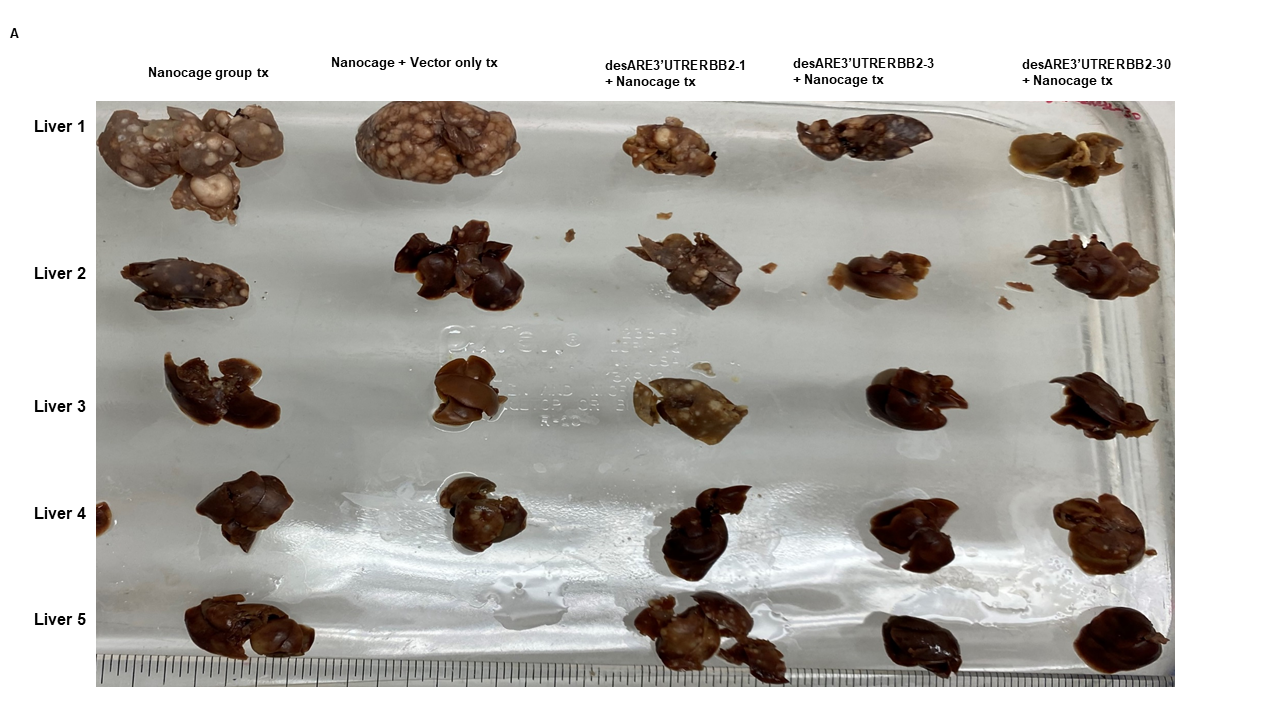

Supplement: Supplementary Figure 2 — Gross pathology of the livers of the control and treated groups (A) Images show the gross pathology of livers in the controls and treated groups with visible hepatomegaly and large metastatic liver nodules in the controls. [file Image_2.tif]

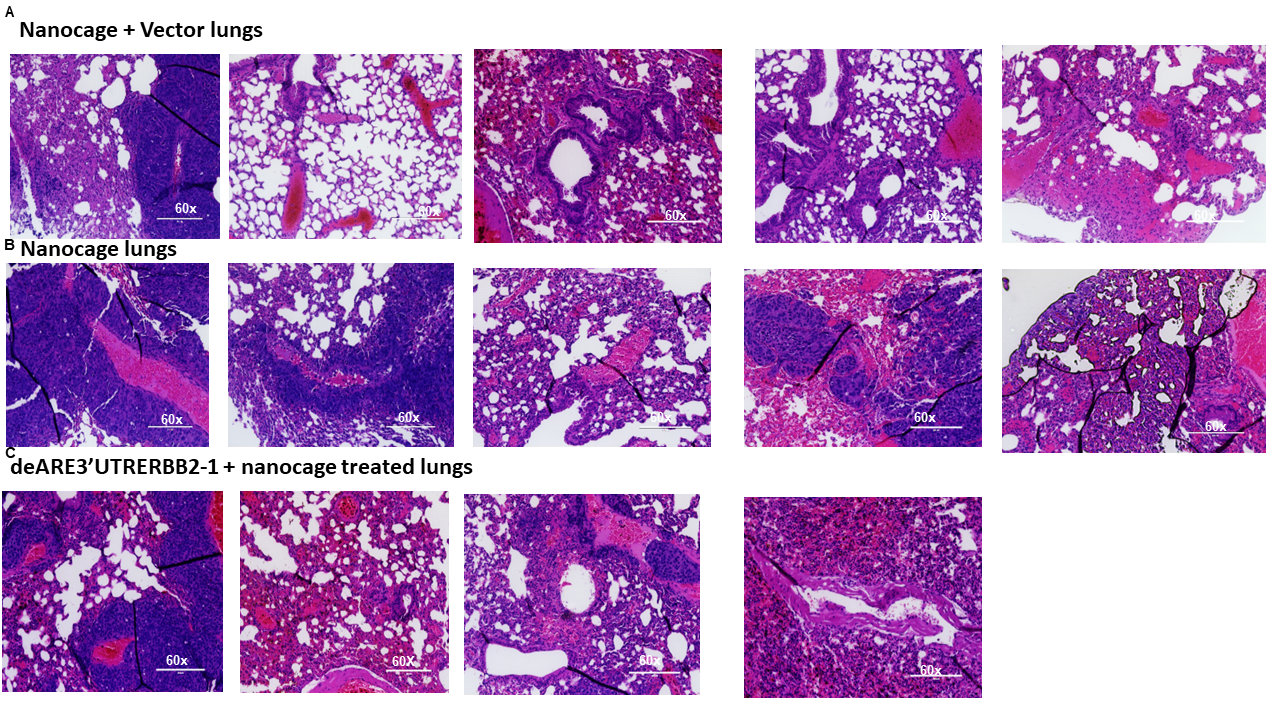

Supplement: Supplementary Figure 3 — H& E staining of the lung tissues from control and treated groups. (A-C) Panel of images shows the H & E staining of the lung tissues from the IO-nanocage only, IO-nanocage+ vector and desARE3’UTRERBB2-1 + IO-nanocages. [file Image_3.tif]

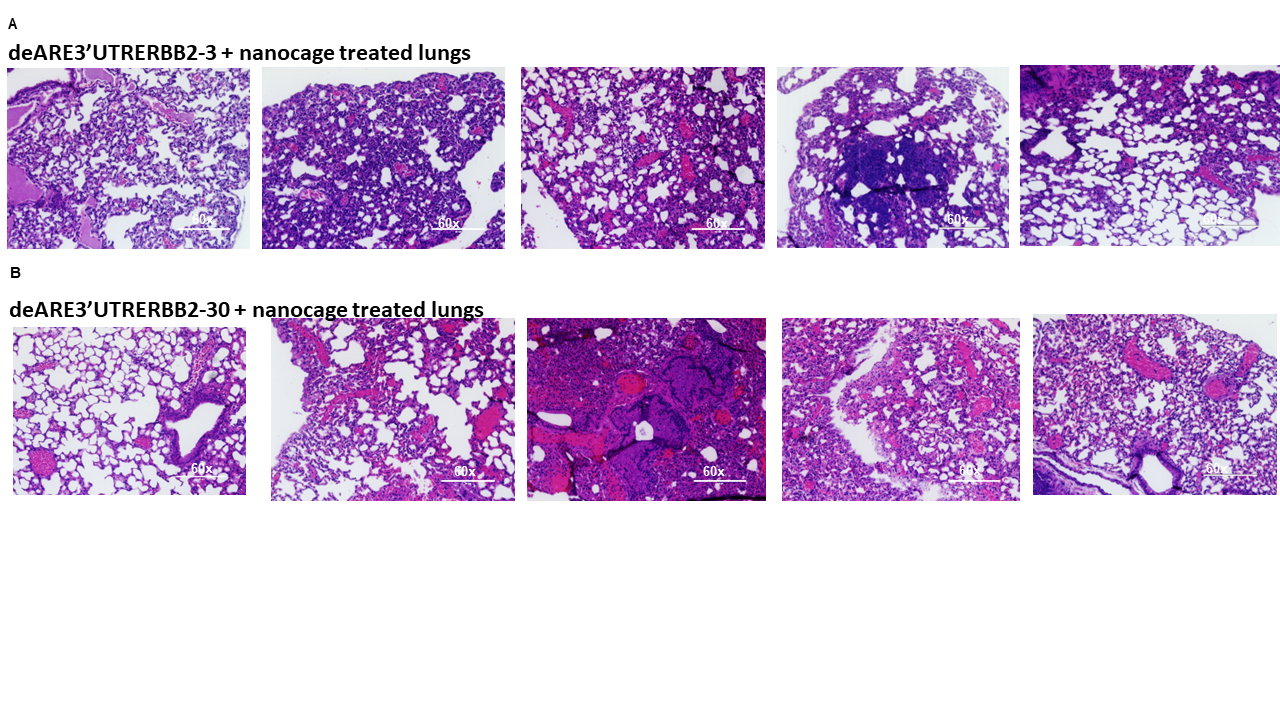

Supplement: Supplementary Figure 4 — H& E staining of the lung tissues from the desARE3’UTRERBB2-3 and 30 treated groups. (A, B) Panel of images shows the H&E staining of the lung tissues from the treated groups of desARE3’UTRERBB2-3 + IO-nanocages and desARE3’UTRERBB2-30 + IO-nanocages. [file Image_4.tif]

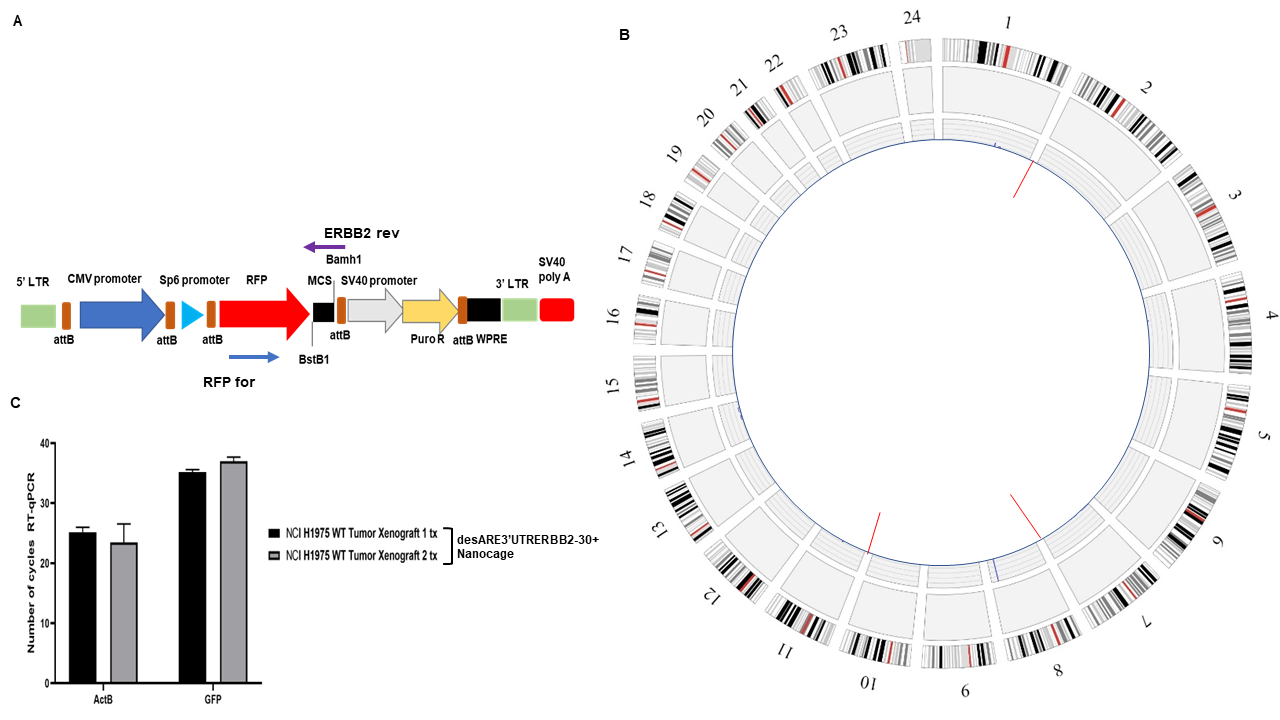

Supplement: Supplementary Figure 5 — Identification of genomic locations of the destabilized 3’UTRERBB2. (A) The schematic representation of the vector with the constructs and arrows (purple) represents the ERBB2 reverse primers and blue the RFP forward primers. (B) The circos plot shows the genomic localization of the engineered destabilized 3’UTR of ERBB2 on the intergenic regions of the chromosomes 1, 7 and 10. C. Bar charts show the qPCR amplification cycle of the house keeping gene ActB in comparison with the RFP in the vector in two tumors treated with the desARE3’UTRERBB2-30. [file Image_5.tif]

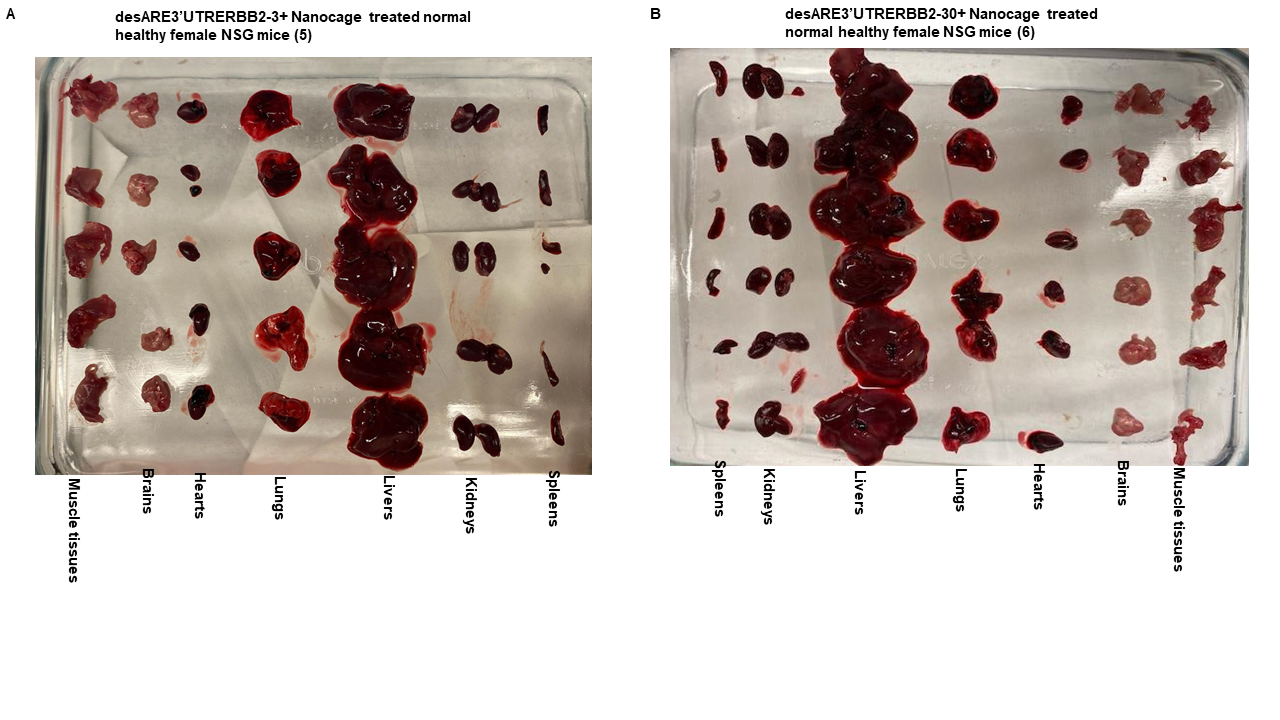

Supplement: Supplementary Figure 6 — Gross pathology images of the normal healthy female NSG mice (i.e., non-tumor bearing mice) treated with the desARE3’UTRERBB2-3 and 30 for 3 months. (A, B) Panel of images shows the gross images of the muscles, brains, livers, lungs, hearts, kidneys and spleens treated with the engineered destabilized 3’UTRERBB2-3 and 30. [file Image_6.tif]

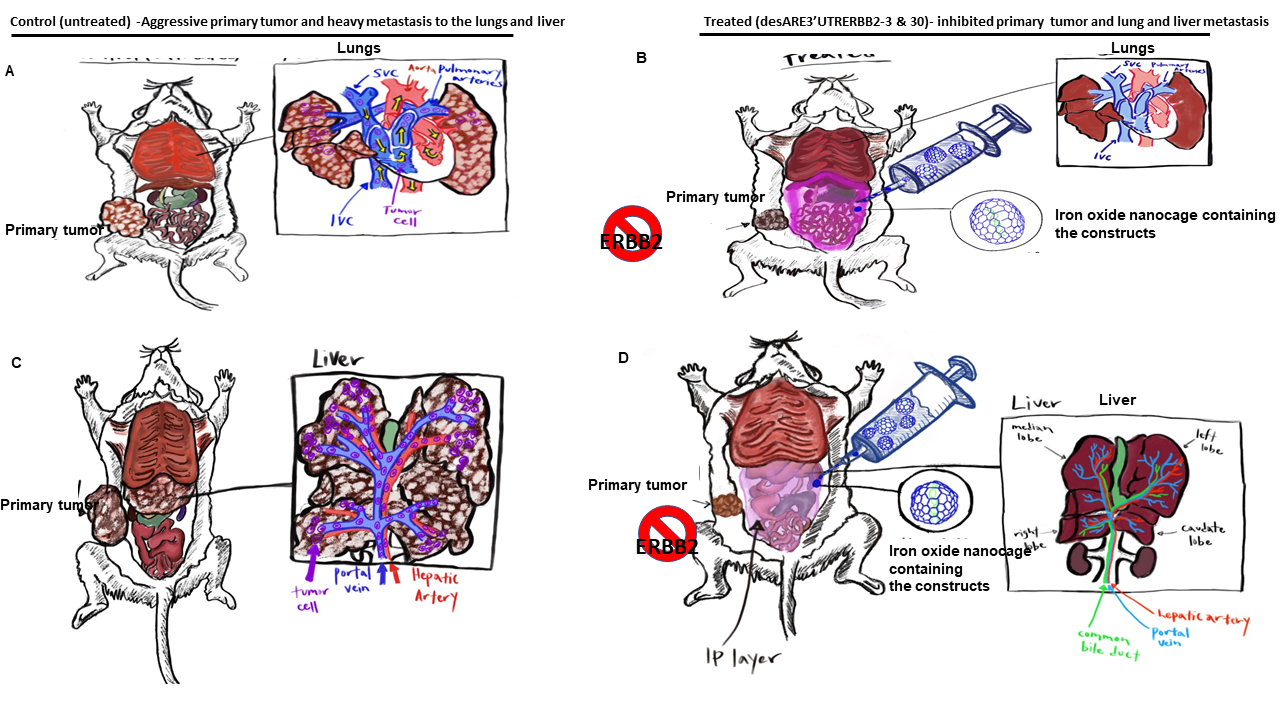

Supplement: Supplementary Figure 7 — Dose response curve showing the viability dose dependent titration of desARE3’UTRERBB2-30 in Osimertinib resistant NSCLC HCC827 and NCIH460 in comparison with the standard of care trastuzumab deruxtecan. (A) Graph shows drug dose response of curve desARE3’UTRERBB2-30 and trastuzumab deruxtecan in NSCLC HCC827 and NCIH460. [file Image_7.tif]

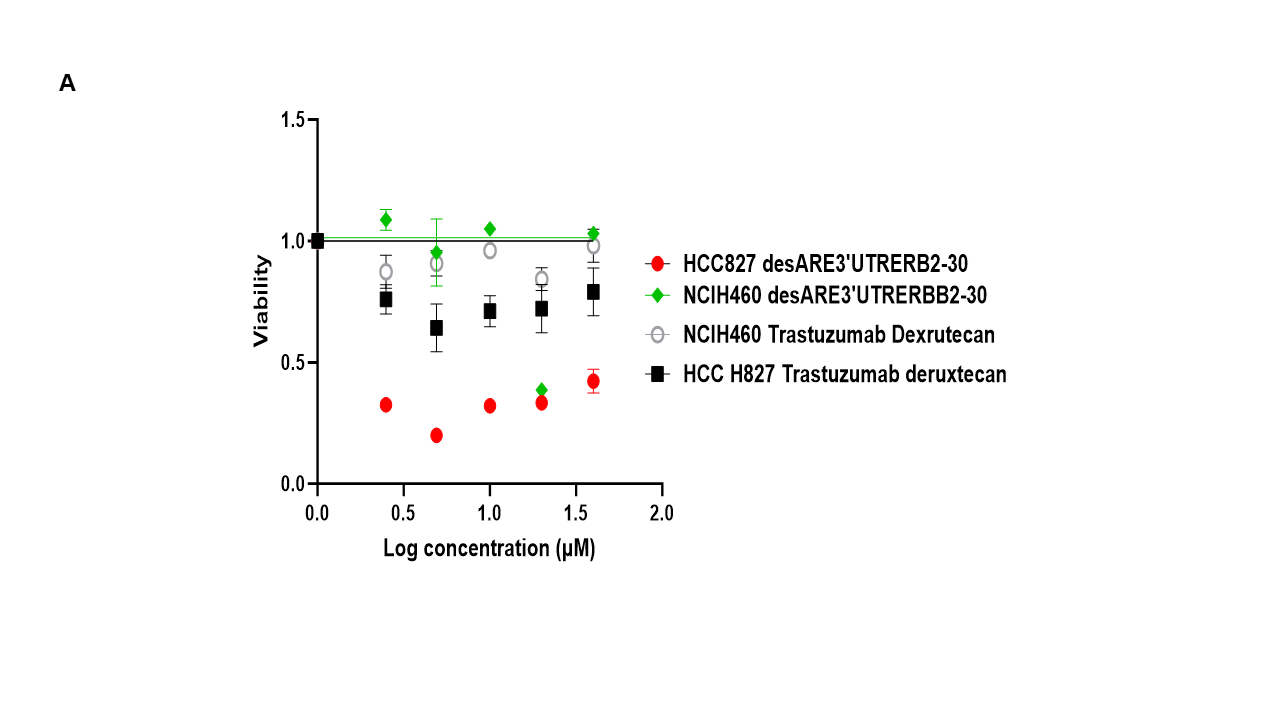

Supplement: Supplementary file 8 [file Image_8.tif]
